# Emerging role of epithelial-mesenchymal transition in hepatic cancer

**DOI:** 10.1186/s13046-016-0419-7

**Published:** 2016-09-13

**Authors:** Go J. Yoshida

**Affiliations:** 1 Department of Pathological Cell Biology, Medical Research Institute, Tokyo Medical and Dental University, 1-5-45 Yushima, Bunkyo-ku, Tokyo, 113-8510 Japan; 2 Japan Society for the Promotion of Science, 5-3-1 Kojimachi, Chiyoda-ku, Tokyo, 102-0083 Japan

**Keywords:** Cancer stem-like cells, Cirrhosis, Epithelial-mesenchymal transition, Hepatic cancer, Matrix metalloproteinases, Wnt/beta-catenin signal

## Abstract

Accumulating evidence suggests that the phenomenon of epithelial-mesenchymal-transition (EMT) plays a fundamental role in the tumor development. Several research articles have been published from Journal of Experimental and Clinical Cancer Research (JECCR) which have investigated into the molecular machineries underlying the importance of EMT for hepatic cancer. Given those recent publications by JECCR, this commentary focuses on the pathological significance of EMT for liver tumor.

## Commentary

Epithelial-mesenchymal transition (EMT) of cancer cells indicates the process in which epithelial cells lose their cell-cell adhesions and apicobasal polarity but also acquire more mesenchymal and invasive/metastatic phenotype [1]. While epithelial-typed proteins characterized by E-cadherin and cytokeratins are down-regulated, mesenchymal markers such as vimentin and N-cadherin are up-regulated. These genetic expression changes are regulated by EMT-inducing transcriptional factors (TFs) such as Snail, Slug, Twist, and ZEB1/2 [1]. Twist and Snail are regulated independently and act cooperatively to promote EMT of hepatocellular carcinoma (HCC) [2]. Furthermore, EMT is known to generate stromal-like cancer cells which acquire the invasion potential into the extracellular matrix mediated by matrix metalloproteinases (MMPs) expression. Xie et al. reported that the discoidin domain receptor 2 (DDR2) up-regulates MT1-MMP and MMP2 depending on ERK2/SNAIL1 axis in HCC [3].

Liver cancer includes primary hepatic neoplasms, which are distinct from the perspectives of both histological and molecular pathologies. Hepatic cancer is mainly classified into HCC, intrahepatic bile duct carcinoma (cholangiocarcinoma), and hepatoblastoma. HCC is the most prevalent among these heterogeneous kinds of hepatic cancer tissues. Chronic infection with hepatitis B and C virus, long-term alcohol consumption, aflatoxin-B1-contaminated food and obesity are the major causes of a late-stage of hepatic fibrosis, also referred to as cirrhosis [4].

Cirrhosis, composed of numerous regenerative nodules, leads to the development of HCC. It is sure that the regenerative nodules at the early stage are benign in nature. However, they are increasingly likely to transform into the dysplastic nodules, which correspond to the pre-malignant conditions [4]. Hepatocytes generally harbor the rapid cellular proliferation under both normal and inflammatory conditions. The rapid rate of cellular division under pathological conditions such as cirrhosis predisposes hepatic stem cells or normal proliferative hepatocytes to accumulate genetic and epigenetic alterations. Still, it remains to be unknown whether the origin of HCC is hepatic stem cells or differentiated hepatocytes.

The molecular analysis of human HCC specimens has elucidated many genetic and epigenetic changes characterized by TP53, beta-catenin, E-cadherin, MET and its ligand hepatocyte growth factor, ErbB receptor family members, and cyclooxygenase 2 (COX2) [4]. Beta-catenin is a crucial downstream TF of the canonical Wnt signal pathway. When Wnt signal pathway is activated, Axin and adenomatous polyposis coli (APC) no longer bind to beta-catenin, resulting in the stabilization and the intra-nuclear translocation of the dissociated beta-catenin. Increased nuclear localization of beta-catenin has been frequently detected in human HCC samples [4, 5]. Wnt/beta-catenin signaling pathway regulates several genes governing cancer-relevant processes, including MYC, cyclin D1, COX2, and MMP7 [5]. Notably, Tang et al. have recently identified CTNND1 (delta-catenin) as a novel oncogene in HCC. The expression level of CTNND1 is positively correlated with invasiveness in human HCC specimens, and further, activates Wnt/beta-catenin signaling. Knockdown of CTNND1 expression surprisingly leads to mesenchymal-epithelial transition (MET), while overexpression results in EMT and enhances metastatic potential of HCC [6].

Du et al. revealed that Brachyury, which is highly conserved through evolution, promotes EMT of HCC via activation of Akt/mTOR signal pathway [7]. Brachyury belongs to the TFs within T-box and contributes to the formation of the midline of a bilaterian organism. Brachyury is expressed not only in the progenitor population throughout somitogenesis but also multiple types of human cancer such as lung and liver. Brachyury has been identified as an essential EMT regulator altering the expression level of E-cadherin and Slug [7]. In particular, there is the positive feedback machinery between Brachyury and TGF-beta in mesenchymal-like tumor cells, which have undergone EMT and up-regulated Brachyury [8]. Brachyury-overexpressed HCC cell lines undergo EMT with Akt activation, therefore promoting the metastasis, particularly the process of extravasation, colonization and the proliferation at the pre-metastatic niche [7].

Remarkably, EMT also contributes to the increased population of cancer stem-like cells (CSCs), which corresponds to the highly tumorigenic subpopulation of tumor cells existing at the top of the hierarchical tumor cell society [1, 9]. The enhanced emergence of CSCs due to EMT is expected to be responsible for tumor heterogeneity and therapeutic resistance [9]. For instance, Zhang et al. have recently demonstrated that the resistance to FOLFOX, the conventional anti-HCC chemotherapeutic regimen, is positively correlated with nestin expression, which leads to EMT and the increased number of CSCs associated with Wnt/beta-catenin activation [10]. Collectively, accumulating evidence has strongly suggested that the emerging roles of potentially therapeutic targets should be regarded as “silver bullets” against difficult-to-cure HCC cases.

## Abbreviations

APC: Adenomatosis polyposis coli
COX2: Cyclooxygenase 2
CSCs: Cancer stem-like cells
DDR2: Discoidin domain receptor 2
EMT: Epithelial-mesenchymal transition
HCC: Hepatocellular carcinoma
MET: Mesenchymal-epithelial transition
MMPs: Matrix metalloproteinases
TF: Transcriptional factor
